# Inefficient Immune Killer Cells Abet HIV Infection

**DOI:** 10.1371/journal.pbio.0040114

**Published:** 2006-03-14

**Authors:** Liza Gross

Viruses employ many strategies to elude their host's defenses, but it's tough to imagine a better tactic than targeting the immune system's communications director. HIV infects and kills helper T lymphocytes (also called CD4 T cells), the white blood cells responsible for mobilizing the mediators of an immune response. Infection results in high casualties among these cells, with half of the infected CD4 T cell population dying every 12 hours. It's thought that the response of another type of T cell—the cytotoxic T lymphocyte (CTL)—may be responsible for a large proportion of these deaths because CTLs recognize and kill infected cells. Based on this assumption, current HIV vaccine strategies are focusing on the CTL response. But it's unclear whether CTL activity or other mechanisms manage these killings, or how much ultimate control the CTL response exerts over the virus.

Evaluating the importance of the CTL response in patients with HIV has proven difficult, yielding conflicting results. In a new study, Becca Asquith, Angela McLean, and their colleagues turned to mathematical modeling to measure the overall effect of the CTL response on the lifespan of infected cells in patients with HIV. Applying different metrics to 28 independent datasets spanning 14 years, the researchers found that CTLs kill roughly 10 million infected cells a day—but, surprisingly, this represents just a fraction of infected cell deaths.

When a virus infects a cell, viral proteins get chopped up into short fragments called epitopes that bind to human leukocyte antigen (HLA) class I molecules and are presented on the infected cell surface. Viral epitopes bound to the HLA molecules act as a signal to the CTL immune response, informing it that the cell is infected and needs to be killed. Patients with HIV born with different forms of these HLA genes experience different rates of progression to AIDS. HIV can develop mutations in its epitopes that allow the virus to escape detection by CTL, often by abrogating HLA binding. The observation that these escape variants outgrow the wild type is much cited evidence that the CTL response exerts selective pressure on the virus.

Asquith et al. have taken this argument one step further and quantified the rate at which escape variants grow out in order to estimate the CTL selective pressure. They developed a model of infected cell dynamics to quantify the rate of escape and fitness cost of escape mutations (since some mutations that confer escape may impair viral replication)—and, thus, the rate at which the CTL response kills virus-infected cells in vivo.

Using published data on 21 escape variants from 12 patients with HIV, the authors estimated each variant's rate of escape, and found “remarkably consistent” results: the rate of escape was very low—less than 0.1 day ^−1^—for 95% of the variants. (This means that it would take an escape variant about five months to outgrow and replace the wild-type population.) The researchers showed that this low escape rate did not occur because the escape variant carried a high fitness cost. They determined this by estimating viral reversion rates—the rate at which an escape variant reverts to wild type after infecting a patient lacking the selective HLA allele—in seven datasets collected from five patients. From these results, Asquith et al. calculated the average rate of escape and reversion for an escape variant to estimate the rate of cell death induced by a single CTL response. Just 2% of infected cell death could be assigned to CTL responses to a single epitope. With indications that a patient with HIV averages between 14 and 19 CTL responses, it's possible as many as 20% of infected cells are killed by CTLs. Thus, while CTLs play an important part in controlling infection, other factors mediate the majority of cell death.

This result helps explain the conflicting findings about the importance of the CTL response in fighting infection—that specific HLA class I alleles are associated with delayed AIDS onset, yet infected cell death rates are not reduced in patients with advanced disease (which suggests that an impaired CTL response does not underlie disease progression). It may be that the modest contributions of the CTL response simply weren't detected in overall cell death rates. Indeed, using the authors' more sensitive approach of only quantifying infected cell death attributable to CTL (rather than total infected cell death), a significant weakening of the immune response with disease progression could clearly be seen. More importantly, these results suggest that unless vaccines based on the CTL pathway are formulated to be far more efficient than the natural response, they are not likely to prevent or clear HIV infection—but it's possible that a CTL-based vaccine might control the number of infected cells and help postpone the onset of symptoms.

## 

**Figure:**
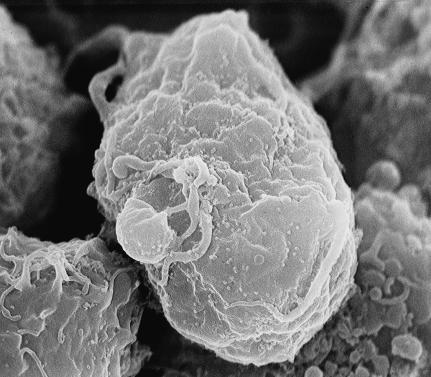
The cytotoxic, "killer," T lymphocyte response kills only a fraction of the total number of HIV-infected lymphocytes in humans infected with the virus. The small spheres visible on the cell surface are viral particles. (Image: C. Goldsmith, CDC)

